# Physicochemical properties and penetration into dentinal tubules of calcium hypochlorite with surfactants

**DOI:** 10.1590/0103-6440202204567

**Published:** 2022-04-29

**Authors:** Hernán Coaguila-Llerena, Julia da Silva Toledo, Ana Paula Ramos, Gisele Faria

**Affiliations:** 1 Department of Restorative Dentistry, São Paulo State University(UNESP), School of Dentistry, Araraquara, SP, Brazil; 2 Department of Chemistry, Ribeirão Preto College of Philosophy Sciences and Letters, São Paulo University(USP), Ribeirão Preto, SP, Brazil

**Keywords:** Calcium hypochlorite, Dentin permeability, Endodontics, Sodium hypochlorite, Surface-active agents

## Abstract

The aim was to assess the physicochemical properties and the penetration into dentinal tubules of calcium hypochlorite solution [Ca(OCl)_2_], with or without surfactants. The surfactants benzalkonium chloride, cetrimide, Tween 80 and Triton X-100 were mixed at different concentrations with sodium hypochlorite solution (NaOCl), Ca(OCl)_2_ and distilled water (control). Once the critical micellar concentration (CMC) of the surfactants in Ca(OCl)_2_ and NaOCl was determined, pH, free chlorine, surface tension and free calcium ions were evaluated. The penetration into dentinal tubules of NaOCl and Ca(OCl)_2_, with or without benzalkonium chloride and Triton X-100 [surfactants that promoted the lowest surface tension of Ca(OCl)_2_], was assessed using human premolars stained with crystal violet. The statistical tests were one-way ANOVA and Tukey’s post-test, Kruskal-Wallis and Dunn’s post-test, two-way ANOVA and Bonferroni’s post-test, and t-test; depending on the assay. The addition of surfactants reduced the surface tension of NaOCl and Ca(OCl)_2_, and did not alter the pH or the free available chlorine of either solution. The addition of all surfactants increased the availability of free calcium ions in Ca(OCl)_2_, especially benzalkonium chloride. Ca(OCl)_2_ exhibited lower penetration into dentinal tubules than NaOCl, and the addition of surfactants did not improve the penetration of Ca(OCl)_2_, but did increase the penetration of NaOCl. It can be concluded that the addition of surfactants to Ca(OCl)_2_ did not increase the penetration into dentinal tubules, but it did promote lower surface tension, without changing the pH or free available chlorine values, and higher availability of free calcium ions in Ca(OCl)_2_.

## Resumo

O objetivo foi avaliar as propriedades físico-químicas e a penetrabilidade nos túbulos dentinários da solução de hipoclorito de cálcio [Ca(OCl)_2_], com ou sem surfactantes. Os surfactantes cloreto de benzalcônio, cetrimida, Tween 80 e Triton X-100 foram misturados em diferentes concentrações com a solução de hipoclorito de sódio (NaOCl), Ca(OCl)_2_ e água destilada (controle). Uma vez determinada a concentração micelar crítica (CMC) dos surfactantes em Ca(OCl)_2_ e NaOCl, foram avaliados o pH, cloro livre, tensão superficial e íons de cálcio livre. A penetrabilidade nos túbulos dentinários de NaOCl e Ca(OCl)_2_, com ou sem cloreto de benzalcônio e Triton X-100 [surfactantes que promoveram a menor tensão superficial de [Ca(OCl)_2_] foi avaliada utilizando pré-molares humanos corados com cristal violeta. Os testes estatísticos foram ANOVA de uma via e pós-teste de Tukey, Kruskal-Wallis e pós-teste de Dunn, ANOVA de duas vias e pós-teste de Bonferroni, e teste t; dependendo do ensaio. A adição de surfactantes reduziu a tensão superficial do NaOCl e Ca(OCl)_2_, e não alterou o pH ou cloro livre das soluções. A adição de todos os surfactantes aumentou a disponibilidade de íons de cálcio livre de Ca(OCl)_2_, principalmente o cloreto de benzalcônio. Ca(OCl)_2_ apresentou menor penetrabilidade nos túbulos dentinários do que NaOCl, e a adição de surfactantes não aumentou a penetrabilidade de Ca(OCl)_2_, mas aumentou a penetrabilidade de NaOCl. Pode-se concluir que a adição de surfactantes no Ca(OCl)_2_ não aumentou a penetrabilidade nos túbulos dentinários, mas promoveu menor tensão superficial, sem alterar os valores de pH ou cloro livre, e maior disponibilidade de íons de cálcio livre em Ca(OCl)_2_.

## Introduction

Sodium hypochlorite solution (NaOCl) is the most commonly used endodontic irrigant in clinical practice ^1^. In water, NaOCl is dissociated in sodium hydroxide and hypochlorous acid. In this aqueous solution, hypochlorous acid partially dissociates into the hypochlorite anion. The free available chlorine is the sum of the hypochlorous acid and hypochlorite anion concentrations in the solution ^2^. Studies have demonstrated that higher values of free available chlorine are associated to a high antimicrobial activity ^3^, and organic tissue dissolution ^2^.

In recent years, calcium hypochlorite solution [Ca(OCl)_2_] has been recommended as an endodontic irrigant, because it can dissolve organic tissue ^4^, and because of its antimicrobial effect ^5^ *.* Additionally, Ca(OCl)_2_ is less cytotoxic than NaOCl ^6^.

It has been suggested that high surface tension may hinder irrigant penetration into dentinal tubules, isthmuses and anatomical irregularities, resulting in reduced antibacterial effectiveness ^7^. For this reason, the penetration into dentinal tubules of NaOCl combined with surfactants [substances that reduce the surface tension] has been previously investigated, with favorable results found in some studies ^8^ ^,^ ^9^. Benzalkonium chloride, cetrimide, Tween 80 and Triton X-100 surfactants have already been combined with NaOCl ^1^ ^,^ ^10^ ^,^ ^11^.

The saturation point of a surfactant in any solution is called the critical micellar concentration (CMC); the concentration of a surfactant above this point does not decrease the surface tension significantly, and leads to the formation of micelles ^10^. It is important to determine the CMC of a surfactant in any solution, because the best wettability properties of the solution are obtained at the calculated concentration ^10^, and because the behavior of the surfactant molecules is different in the presence of micelles ^12^. Although the CMC of benzalkonium chloride, Tween 80 and Triton X-100 in 2.4% NaOCl has been previously reported ^10^ ^,^ ^11^, there is no available literature on the CMC of these surfactants in 2.5% Ca(OCl)_2_, or on the CMC of cetrimide in either 2.5% NaOCl or 2.5% Ca(OCl)_2._

In the study by Iglesias et al. ^13^, benzalkonium chloride and cetrimide were added to Ca(OCl)_2_ at a concentration equal to that of the CMC of NaOCl. Both surfactants reduced the surface tension of 2.5% NaOCl and 2.5% Ca(OCl)_2_, and did not alter their pH, free available chlorine, or pulp dissolution properties. Nevertheless, there is no available literature on the penetration into dentinal tubules of Ca(OCl)_2_, with or without surfactants. Likewise, there is no available literature on Tween 80 and Triton X-100 in combination with Ca(OCl)_2_.

Therefore, the present study aimed to assess the physicochemical properties as well as the penetration into the dentinal tubules of 2.5% Ca(OCl)_2_, with or without surfactants at CMC, compared with 2.5% NaOCl. The null hypothesis was that there would be no difference among these solutions with or without the surfactants, regarding physicochemical properties of pH, free available chlorine, free calcium ions as well as penetration into the dentinal tubules.

## Methodology

### Preparation of irrigation solutions, and determination of surface tension and CMC

The stock Ca(OCl)_2_ and NaOCl were prepared at approximately 6% weight/volume (w/v). The Ca(OCl)_2_ was prepared by diluting calcium hydroxide powder (Êxodo Científica, Sumaré, SP, Brazil) in distilled water under constant agitation for 30 minutes, and then filtering the solution using filter paper to remove the sediment. The NaOCl was prepared by diluting a 10% NaOCl (AraQuímica, Araraquara, SP, Brazil) in distilled water. Then, the free available chlorine of both stock solutions was determined by using the iodine/sodium thiosulfate titration method, and the solutions were stored in a refrigerator for 20 days at most until use, at 4°C, protected from light. For the evaluation of the physicochemical properties, the solutions were taken from the refrigerator and kept in the room until the temperature of the solutions equaled the room temperature determined for each assay. The 2.5% Ca(OCl)_2_ and 2.5% NaOCl, with or without benzalkonium chloride, cetrimide, Tween 80 and Triton X-100 (Sigma-Aldrich, St. Louis, MO, USA), were prepared from stock solutions at different concentrations (Table 1).

**Table 1:** Concentration range of benzalkonium chloride, cetrimide, Tween 80 and Triton X-100, used to determine the critical micellar concentration (CMC) in water, in 2.5% sodium hypochlorite solution (NaOCl) and in 2.5% calcium hypochlorite solution [Ca(OCl)_2_]

Solution	Concentration of surfactant (%)
Water - benzalkonium chloride	0.001 - 2
Water - cetrimide	0.01 - 1
Water - Tween 80	0.000393 - 3
Water - Triton X-100	0.00312 - 3
2.5% NaOCl - benzalkonium chloride	0.001 - 1
2.5% NaOCl - cetrimide	0.0005 - 1
2.5% NaOCl - Tween 80	0.003 - 1
2.5% NaOCl - Triton X-100	0.00007 - 1
2.5% Ca(OCl)_2_ - benzalkonium chloride	0.001 - 0.3
2.5% Ca(OCl)_2_ - cetrimide	0.0001 - 0.2
2.5% Ca(OCl)_2_ - Tween 80	0.003 - 1
2.5% Ca(OCl)_2_ - Triton X-100	0.00007 - 1

The surface tension of all the combinations (n = 3) was measured by using the pendant-drop method at 22°-24°C room temperature. Each solution was placed in a syringe coupled to an OCA-20 system (DataPhysics Instruments, Filderstadt, Germany), wherein it formed a drop digitally captured by a charge-coupled device. The surface tension was then calculated automatically based on the drop shape, using a SCA-20 software program (DataPhysics Instruments), as previously described ^9^. Next, the surface tension data were plotted in an Origin 8 software program (OriginLab, Northampton, MA, USA), and a linear regression of the curve was applied on both the “x” (surfactant concentration) and the “y” axes (surface tension). The intersection of the two lines (resulting from the “x” and “y” axes) allowed calculating the CMC, and determining the surface tension at CMC. The 2.5% NaOCl and the 2.5% Ca(OCl)_2_, mixed with the surfactants at CMC, were used to assess pH, free available chlorine and free calcium ions.

### Determination of pH

The freshly prepared 2.5% NaOCl and 2.5% Ca(OCl)_2_, with and without surfactants at CMC (n = 3), were stirred. Then, the pH of each solution was measured using a pH-meter (DM-22, Digimed, São Paulo, SP, Brazil) at 22°C room temperature, according to the requirements of the European Pharmacopoeia.

### Determination of free available chlorine

The free available chlorine was determined by using the iodine/sodium thiosulfate titration method (n = 3). A total of 10 mL of the diluted 2.5% NaOCl and 2.5% Ca(OCl)_2_ (5g each in 100 mL of distilled water), with or without surfactants at CMC, was mixed with 30 mL of 5% potassium iodide (Êxodo Científica), and with 10 mL of 99.8% acetic acid (Neon Comercial, Suzano, SP, Brazil). Afterwards, a previously standardized 0.1 N sodium thiosulfate solution (Labsynth Produtos Para Laboratórios, Diadema, SP, Brazil) was dripped into the NaOCl and Ca(OCl)_2_ using a standard 50 mL burette, until they became pale yellow. Immediately, a 0.5% starch solution (Êxodo Científica) was added, making the solution an intense blue colour. Subsequently, 0.1 N sodium thiosulfate (Labsynth) was dripped into the blue solution until it became transparent. The required sodium thiosulfate volume was recorded ^10^. The room temperature was maintained at 20°C ^14^. The data were expressed as a percentage (%) of w/v.

### Determination of free calcium ions

The concentration of free calcium ions in the 2.5% Ca(OCl)_2_ with or without surfactants at CMC (n = 3) was determined at 22°-24°C room temperature by potentiometry, using a calcium-selective electrode. The potentiometer (Bante Instruments, Sugar Land, TX, USA) allowed measuring free calcium ions conductivity (expressed as a mmol/L concentration) using a calibration curve (R^2^=0.9996) taken from a standard calcium solution (0.1 M). Then, 10 µL to 40 µL of the solutions were added sequentially to 20 mL of distilled water, and an electrode was placed at each addition to obtain the measurements. The expected free calcium ions was calculated at each addition of the 20 mL of distilled water plus 10 µL to 40 µL of the experimental solutions. This theoretical calculation was performed considering that the formula for Ca(OCl)_2_ has 2 moles of hypochlorite ion for 1 mole of calcium ion.

### Penetration into dentinal tubules

This assay was performed by selecting the benzalkonium chloride and Triton X-100 surfactants, which promoted the lowest surface tension of both 2.5% NaOCl and 2.5% Ca(OCl)_2_. The sample size was calculated using the G* Power 3.1.7 software program for Windows (Heinrich-Heine-Universität Düsseldorf, Germany). The calculation was based on an effect size = 0.44 (based on a pilot study), test power (β) = 0.8, and α = 0.05, using the F-test family for one-way analysis, and showed that 72 specimens (n = 12) were required.

After approval of the study by the Ethics Committee of the School of Dentistry (CAAE: 09799019.4.0000.5416), 75 freshly extracted human, permanent, single-rooted premolars donated by the tooth bank were scraped of any residual tissue tags, disinfected in 2.5% NaOCl for 5 min, and stored in 0.1% thymol at 4°C until use. The exclusion criteria comprised teeth with more than one root canal / apical foramen, oval-shaped canals, previous treatment, calcification, internal / external resorption, cracks, fractures on the root surface, > 5° Schneider angle, or canals that allowed the insertion of a file exceeding an ISO size 15 K-file into the apical foramen. To confirm the inclusion criteria, the teeth were examined using a stereomicroscope (Leica Microsystems, Wetzlar, Germany) and radiographed using a digital sensor (FONA CDR Elite, Schick by Sirona Dental, Long Island, NY, USA). Radiographic analysis was performed from mesiodistal and buccolingual projections, to select the teeth with similar dimensions, single, round-shaped canals, straight roots (< 5° Schneider angle), and single foramen (Vertucci’s type I configuration). The radiographic images were analyzed using an Image J software program (National Institutes of Health, Bethesda, MD, USA). The root canals were considered round-shaped when the buccolingual diameter equalled the mesiodistal diameter. After selection, the specimens were randomly allocated into six experimental groups (n = 12) using a computer algorithm (http://www.random.org) to ensure homogeneous distribution. The crowns were removed, and the root length was standardized at 16 mm. The root canals were instrumented using the ProDesign Logic system (Easy Equipamentos Odontólogicos, Belo Horizonte, MG, Brazil). At 15 mm working length, the 25.01, 25.03, 25.05 and 40.05 files were used at 350-600 rpm speed and 1-4 Ncm torque, depending on the file, using an electric motor (VDW Silver, VDW, Munich, Germany) ^9^. The root canals were irrigated with 2 mL of 2.5% NaOCl for 1 minute at each instrument change, followed by irrigation with 5 mL of 17% EDTA for 3 minutes, and 5 mL of distilled water. The canals were dried with paper cones equivalent to the last file. Next, the canals were filled with 1% crystal violet solution (Labsynth Produtos para Laboratórios, Diadema, SP, Brazil), and kept at 37°C and 95% relative humidity for 3 days. Then, they were irrigated with 20 mL of distilled water, and the apex was sealed with composite resin to create a closed system ^1^. The experimental groups were distributed as follows: 2.5% Ca(OCl)_2_ + benzalkonium chloride, 2.5% Ca(OCl)_2_ + Triton X-100, 2.5% NaOCl + benzalkonium chloride, 2.5% NaOCl + Triton X-100, 2.5% Ca(OCl)_2_ and 2.5% NaOCl, wherein the benzalkonium chloride and Triton X-100 surfactant groups used a previously determined CMC. Three teeth were irrigated with distilled water to serve as additional controls of the reaction. Next, the specimens were irrigated at 22-24°C room temperature with 5 mL of the irrigating solutions for 2 minutes ^1^ using a 5 mL syringe (Ultradent Products, South Jordan, UT, USA) coupled to a 27 G side-vented needle (Endo-Eze^®^, Ultradent Products), positioned 2 mm short of the working length. The root canals of all the groups were then irrigated with 5 mL of distilled water, and were sectioned transversely along their longitudinal axis at 3, 7 and 12 mm from the apex, using a low speed cutting machine (Isomet 1000, São Paulo, SP, Brazil), to obtain segments from the cervical, middle and apical segments. The cervical surface of each segment was polished using 1000-grit abrasive paper (3M ESPE, St. Paul, MN, USA), under constant irrigation with water. A stereomicroscope (LeicaM80, Leica Microsystems) and the Leica Application Suite EZ 3.0 software program (Leica Microsystems) were used to obtain the images. The penetration depth was measured in micrometers (μm) at 10 equidistant regions using the Image J program (National Institutes of Health, NIH) ^9^. A previously calibrated and blinded examiner performed the measurements twice, with a 2-week interval (intraclass correlation coefficient > 0.9).

### Statistical analysis

The data were analyzed using GraphPad Prism 5 (GraphPad Software, San Diego, CA, USA). An initial screening to assess data normality was performed by using the D'Agostino-Pearson test. The statistical tests used were one-way analysis of variance (ANOVA) and Tukey’s post-test (surface tension, penetration into dentinal tubules - comparison among solutions), Kruskal-Wallis and Dunn’s post-test (penetration into dentinal tubules - comparison among segments, showing no homogeneity of variance), two-way ANOVA and Bonferroni’s post-test (free calcium ions), or the t-test (pH and free available chlorine), at a significance level of 5%.

## Results

### Surface tension and critical micellar concentration

**Figure 1:**
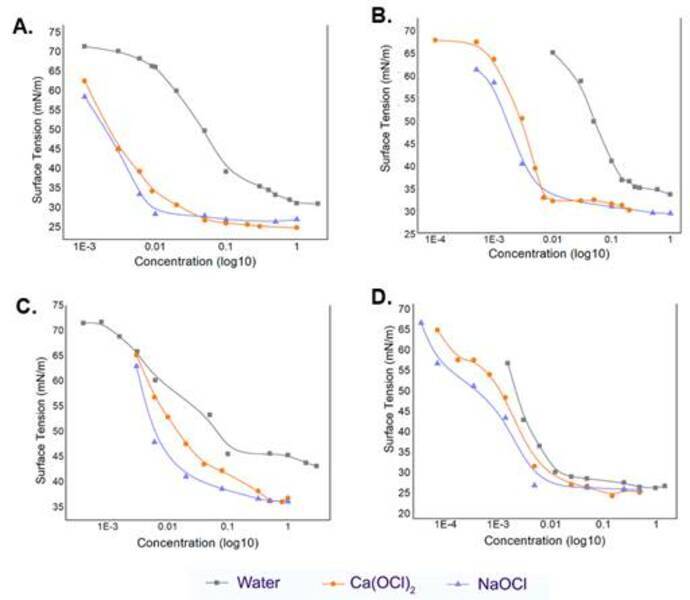
Effect of adding (a) benzalkonium chloride, (b) cetrimide, (c) Tween 80, and (d) Triton X-100 to water, 2.5% calcium hypochlorite solution [Ca(OCl)_2_], and 2.5% sodium hypochlorite solution (NaOCl). The surface tension versus concentration (represented as log10 for better visualization) was used to determine the critical micellar concentration (CMC) of each surfactant in water, Ca(OCl)_2_ and NaOCl.

Figure 1 shows the surface tension of the water, and both the 2.5% NaOCl and 2.5% Ca(OCl)_2_, combined with the surfactants at different concentrations. These data were used to calculate the CMC of the surfactants (Table 2). When the surfactants were combined with water, there was no difference between the CMC values of benzalkonium chloride and cetrimide (p>0.05), and both had a higher CMC values than Tween 80 and Triton X-100 (p<0.05). When combined with 2.5% NaOCl, all the surfactants showed different CMC values (p<0.05), but when combined with 2.5% Ca(OCl)_2_, the benzalkonium chloride and Triton X-100 surfactants showed similar CMC values (p>0.05). The CMC values of benzalkonium chloride and cetrimide were similar in both 2.5% NaOCl and 2.5% Ca(OCl)_2_ (p>0.05), whereas the CMC values of Tween 80 and Triton X-100 were different (p<0.05).

The surface tension of the water, of the 2.5% NaOCl and 2.5% Ca(OCl)_2_, and of all these solutions combined with the surfactants at CMC, are shown in Table 2. Combined with water, Triton X-100 promoted a higher reduction in surface tension, followed by benzalkonium chloride and cetrimide, which were not different from each other (p>0.05), and next by Tween 80 (p<0.05). The 2.5% NaOCl showed higher surface tension than 2.5% Ca(OCl)_2_ (p<0.05). The addition of the surfactants at CMC reduced the surface tension of 2.5% NaOCl and 2.5% Ca(OCl)_2_ (p<0.05). It is important to note that benzalkonium chloride and Triton X-100 promoted the lowest surface tensions in both 2.5% NaOCl and 2.5% Ca(OCl)_2_ (p<0.05). Mostly, 2.5% NaOCl with surfactants showed lower surface tension than 2.5% Ca(OCl)_2_ with surfactants (p<0.05), except in the case of Triton X-100, which promoted a similar reduction in surface tension in both solutions (p>0.05).

**Table 2:** Mean and standard deviation (in parentheses) of critical micellar concentration - CMC (in %) of benzalkonium chloride, cetrimide, Tween 80 and triton X-100in water, 2.5% sodium hypochlorite solution (NaOCl) and 2.5% calcium hypochlorite solution [Ca(OCl)_2_], and the surface tension (in millinewton/meter) of the solutions with the surfactants at CMC.

Solution	Critical micellar concentration (%)	Surface tension (mN/m)
Water	-	72.81 (0.23) ^a^
Water - benzalkonium chloride	0.1066 (0.0019%) ^a^	35.87 (0.13) ^b^
Water - cetrimide	0.1122 (0.0009%) ^a^	36.22 (0.32) ^b^
Water - Tween 80	0.0701 (0.0041%) ^b^	45.89 (0.48) ^c^
Water - Triton X-100	0.0249 (0.0004%) ^c^	29.45 (0.33) ^d^
2.5% NaOCl	-	69.66 (0.16) ^aA^
2.5% NaOCl - benzalkonium chloride	0.0093 (0.0001%) ^aA^	27.90 (0.21) ^bA^
2.5% NaOCl - cetrimide	0.0065 (0.0005%) ^bA^	30.74 (0.12) ^cA^
2.5% NaOCl - Tween 80	0.0221 (0.0006%) ^cA^	38.00 (0.41) ^dA^
2.5% NaOCl - Triton X-100	0.0051 (0.0004%) ^dA^	27.84 (0.39) ^bA^
2.5% Ca(OCl)_2_	-	68.30 (0.47) ^aB^
2.5% Ca(OCl)_2_ - benzalkonium chloride	0.0098 (0.0003%) ^aA^	29.27 (0.24) ^bB^
2.5% Ca(OCl)_2_ - cetrimide	0.0066 (0.0001%) ^bA^	32.42 (0.18) ^cB^
2.5% Ca(OCl)_2_ - Tween 80	0.0248 (0.0013%) ^cB^	41.92 (0.83) ^dB^
2.5% Ca(OCl)_2_ - Triton X-100	0.0111 (0.0003%) ^aB^	27.63 (0.07) ^eA^

Different lower case letters in the columns indicate significant differences among NaOCl, Ca(OCl)_2_, water and associations with different surfactants (p<0.05). Different capital letters indicate significant differences between isolated 2.5% NaOCl and 2.5% Ca(OCl)_2_, and between 2.5% NaOCl and 2.5% Ca(OCl)_2_ associated with the same surfactant (p<0.05).

### pH and free available chlorine

The addition of a surfactant did not change the pH of the 2.5% NaOCl or the 2.5% Ca(OCl)_2_ (p>0.05). However, 2.5% NaOCl with and without a surfactant showed a higher pH, compared with 2.5% Ca(OCl)_2_ with and without a surfactant (p<0.05). The addition of a surfactant did not alter the free available chlorine of either 2.5% NaOCl or 2.5% Ca(OCl)_2_ (p>0.05), as shown in Table 3.

**Table 3:** Mean and standard deviation (in parenthesis) of physicochemical properties of pH and free available chlorine of 2.5% sodium hypochlorite solution (NaOCl) and 2.5% calcium hypochlorite solution [Ca(OCl)_2_] with and without benzalkonium chloride, cetrimide, Tween 80 and Triton X-100 at critical micellar concentration (CMC)

Solution	pH	Free available chlorine (% w/w)
2.5% NaOCl	12.33 (0.17) ^aA^	2.54 (0.08) ^aA^
2.5% NaOCl - benzalkonium chloride	12.25 (0.02) ^aA^	2.52 (0.06) ^aA^
2.5% NaOCl - cetrimide	12.36 (0.03) ^aA^	2.50 (0.04) ^aA^
2.5% NaOCl - Tween 80	12.42 (0.04) ^aA^	2.56 (0.02) ^aA^
2.5% NaOCl - Triton X-100	12.29 (0.05) ^aA^	2.57 (0.04) ^aA^
2.5% Ca(OCl)_2_	11.76 (0.01) ^bB^	2.61 (0.02) ^aA^
2.5% Ca(OCl)_2_ - benzalkonium chloride	11.76 (0.04) ^bB^	2.55 (0.04) ^aA^
2.5% Ca(OCl)_2_ - cetrimide	11.84 (0.07) ^bB^	2.62 (0.02) ^aA^
2.5% Ca(OCl)_2_ - Tween 80	11.79 (0.05) ^bB^	2.57 (0.04) ^aA^
2.5% Ca(OCl)_2_ - Triton X-100	11.81 (0.04) ^bB^	2.57 (0.04) ^aA^

Different lower case letters in columns indicate significant differences among NaOCl, Ca(OCl)_2_ and associations with different surfactants (p<0.05). Different capital letters indicate significant differences between isolated 2.5% NaOCl and Ca(OCl)_2_, and between 2.5% NaOCl and Ca(OCl)_2_ associated with the same surfactant (p<0.05).

### Free calcium ions

**Figure 2:**
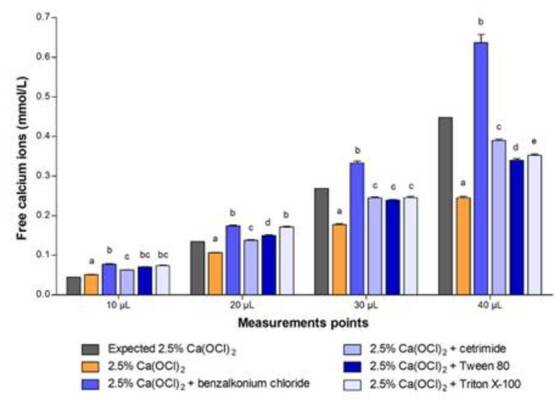
Mean and standard deviation of free calcium ions (mmol/L) of 2.5% calcium hypochlorite solution [Ca(OCl)_2_] in association with benzalkonium chloride, cetrimide, Tween 80 and Triton X-100, at critical micellar concentration.

The use of a calcium-selective electrode allowed determining the availability of free calcium ions in the solution, and the effect of surfactant addition on this parameter. The results are shown in Figure 2. The addition of surfactants to 2.5% Ca(OCl)_2_ increased the availability of free calcium ions, compared with 2.5% Ca(OCl)_2_ without surfactants (p<0.05). In particular, the addition of benzalkonium chloride resulted in higher availability of free calcium ions in 2.5% Ca(OCl)_2_, compared with the other surfactants (p<0.05).

*Different letters in each column indicate a significant difference among the solutions at each measurement point (p<0.05). The expected Ca(OCl)*_*2*_ *values were not included in the statistical analysis.*

### Penetration into dentinal tubules

**Figure 3:**
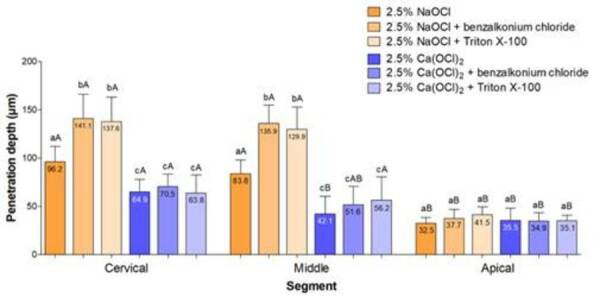
Mean and standard deviation in micrometres (μm) of penetration depth into dentinal tubules of 2.5% sodium hypochlorite solution (NaOCl) and 2.5% calcium hypochlorite solution [Ca(OCl)_2_] combined with benzalkonium chloride or Triton X-100. The mean value is shown in each column.

**Figure 4:**
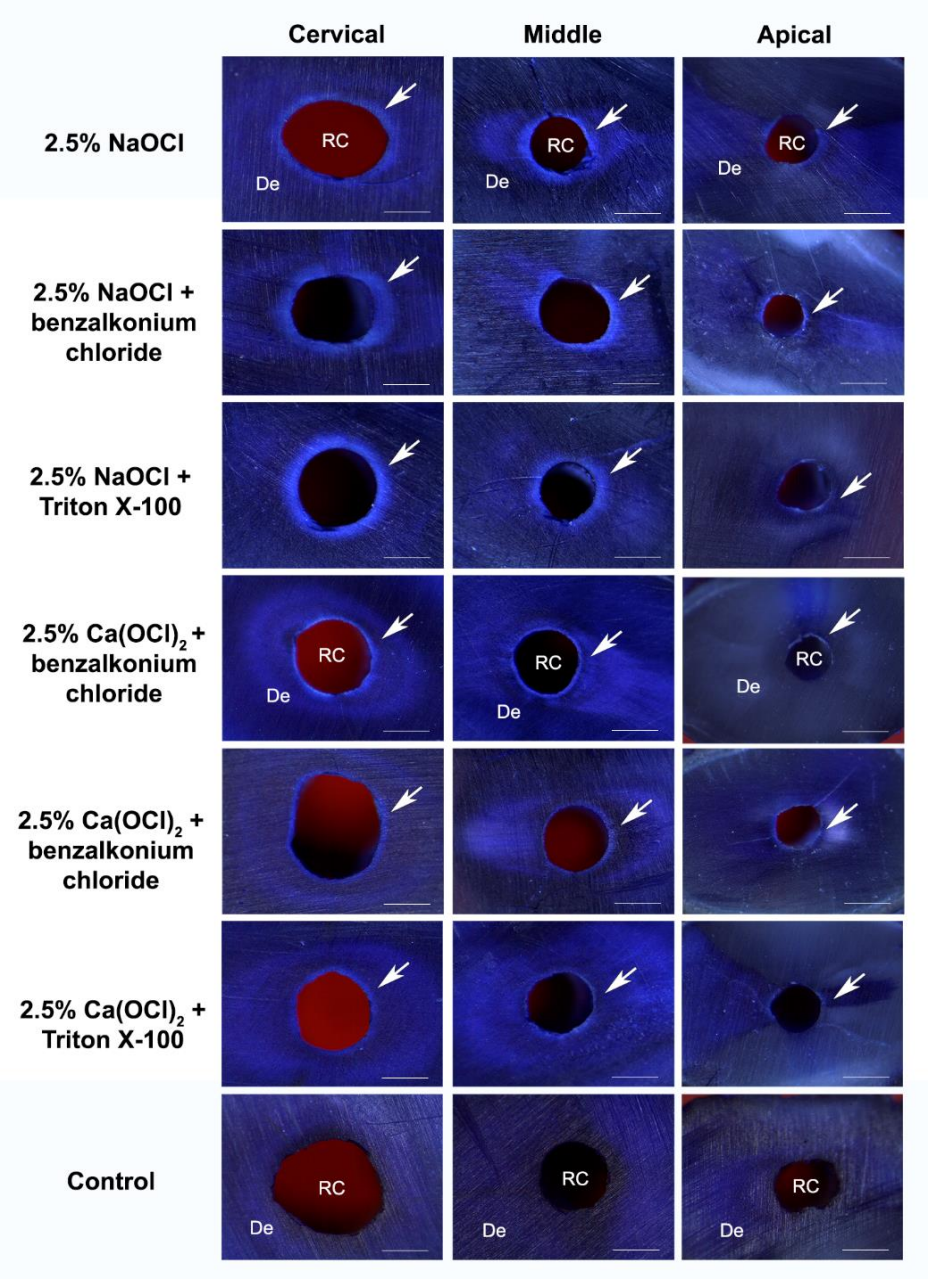
Representative images of penetration depth into dentinal tubules of 2.5% sodium hypochlorite solution (NaOCl) and 2.5% calcium hypochlorite solution [Ca(OCl)_2_] combined with benzalkonium chloride or Triton X-100 in cervical, middle and apical segments. The bleached crystal violet represents the penetration depth of irrigants into the dentine (arrow) (bar = 500 μm). De, dentine; RC, root canal.

The 2.5% NaOCl + benzalkonium chloride and 2.5% NaOCl + Triton X-100 groups had the highest penetration depth in the cervical and middle segments, in comparison with the other groups (p<0.05). The penetration depth of 2.5% Ca(OCl)_2_ was lower than that of 2.5% NaOCl in the cervical and middle segments (p<0.05). In these segments, there were no differences among 2.5% Ca(OCl)_2_, with or without surfactants (p>0.05). In the apical segment, there were no significant differences among any of the groups (p>0.05). Comparison of the segments revealed that the penetration depth in the cervical and middle segments was no different for 2.5% NaOCl with or without surfactants (p>0.05), but it was different for these groups in the apical segment (p<0.05). The 2.5% Ca(OCl)_2_ group had a higher penetration depth in the cervical segment (p<0.05), whereas there were no differences in the middle and apical segments (p>0.05). The 2.5% Ca(OCl)_2_ + benzalkonium chloride group showed no differences between the cervical and middle segments, or between the middle and apical segments (p>0.05). The penetration depth of 2.5% Ca(OCl)_2_ + Triton X-100 group was not different between the cervical and the middle segments, but was lower in the apical versus cervical or middle segments (p>0.05) (Figure 3 and Figure 4).

*Different lowercase letters in columns of each segment indicate a significant difference among the solutions. Different uppercase letters in columns indicate a significant difference for each solution.*

## Discussion

The CMC of the surfactants in 2.5% NaOCl and 2.5% Ca(OCl)_2_, and the surface tension of 2.5% NaOCl and 2.5% Ca(OCl)_2_ with surfactants at CMC was determined in the first part of the study. Then, the physicochemical properties of pH, free available chlorine, and free calcium ions of 2.5% NaOCl and 2.5% Ca(OCl)_2_ with or without surfactants at CMC were evaluated. Based on these initial findings, two surfactants (benzalkonium chloride and Triton X-100) were selected to assess penetration of the irrigants into the dentinal tubules, because they provided the lowest surface tension of 2.5% Ca(OCl)_2_ and 2.5% NaOCl. The null hypothesis was partially rejected, because there were some differences in the parameters for the solutions studied.

It has been reported that Ca(OCl)_2_ forms a white precipitate at room temperature (25°C), even after filtering ^13^ ^,^ ^15^, and that this may affect the equilibrium concentration of the solution ^13^. To minimize the formation of this precipitate, Ca(OCl)_2_ was stored at 4°C until use ^15^. NaOCl was also stored at 4°C until use, to counteract the instability of the solution ^15^.

The mechanism of the irrigating solution combined with the surfactant causes the surfactant to be adsorbed on the liquid/air interface of the solution, leading to a decrease in the surface tension of the solution ^12^ ^,^ ^16^. The adsorbed surface remains in equilibrium with the surfactant in the solution, as the concentration of the surfactant is gradually increased to a saturation point called CMC ^16^. The CMC of benzalkonium chloride and of cetrimide were similar in 2.5% NaOCl and 2.5% Ca(OCl)_2_, whereas the CMC of Tween 80 and Triton X-100 were higher in 2.5% Ca(OCl)_2_. The CMC of benzalkonium chloride in 2.5% NaOCl (0.0093%) was relatively close to the previously reported 0.008% in 2.4% NaOCl ^10^. On the other hand, the CMC of Tween 80 (0.0221%) and Triton X-100 (0.0051%) observed in our study using 2.5% NaOCl was different from the CMC for Tween 80 (0.01%) and Triton X-100 (0.00035%) previously reported by Bukiet et al.^11^ using a 2.4% NaOCl. The differences could be attributed to the high temperature sensitivity of Tween 80 and Triton X-100, both ethoxylated non-ionic surfactants ^12^. In the present study, measurements were performed at 22-24°C room temperature ^9^, while the study by Bukiet et al.^11^ was performed at 37°C. Regarding the CMC of cetrimide in 2.5% NaOCl (0.0065%), there is no available literature concerning this association.

Among the physicochemical properties of chlorinated solutions in endodontics, the pH factor may affect the free available chlorine ^17^, because it is directly related to the dissociation of hypochlorous acid ^2^. In the present study, 2.5% NaOCl and 2.5% Ca(OCl)_2_ were alkaline, as corroborated by previously reported literature ^9^ ^,^ ^13^ ^,^ ^15^, and the addition of surfactants did not affect the pH, which also agrees with previous studies ^8^ ^,^ ^13^. The surfactant did not influence the pH of either the 2.5% NaOCl or the 2.5% Ca(OCl)_2_, because of its low concentration ^13^. However, NaOCl with and without surfactants had a higher pH than the equivalent Ca(OCl)_2_. These results diverge from those of the study by Leonardo et al*.* ^15^, who observed that 2.5% NaOCl and 2.5% Ca(OCl)_2_ showed similar pH at 25°C. The differences could be attributed to the free available chlorine values, since Leonardo et al.^15^ reported that 2.5% Ca(OCl)_2_ had higher free available chlorine values than 2.5% NaOCl, whereas the free available chlorine values found in the present study were similar for both solutions, and very close to what was expected. It has been reported that there is a correlation between low pH and low free available chlorine ^18^; this being the case, the correlation may have influenced the comparison.

The addition of surfactants did not alter the free available chlorine of either 2.5% NaOCl or 2.5% Ca(OCl)_2_, in agreement with previous studies ^10^ ^,^ ^13^. Moreover, 2.5% NaOCl and 2.5% Ca(OCl)_2_ showed similar free available chlorine, as expected, since the solutions were prepared from stock solutions with known free available chlorine values. However, it is important to note that the stock Ca(OCl)_2_ should have had a 6% concentration, but it was actually lower (data not shown); the resulting concentration corroborated a study showing that 5% and 10% Ca(OCl)_2_ both had lower free available chlorine than expected ^4^. It is important to emphasize that one of the aims of this laboratory study was to evaluate the influence of the addition of surfactants in NaOCl and Ca(OCl)_2_ on pH and free available chlorine; however, a clinical extrapolation, is not possible since it has been shown that dentine debris and pulp tissue can affect those properties ^19^ ^,^ ^20^. This may be considered a limitation of the present study.

The addition of surfactants, especially benzalkonium chloride and Triton X-100, significantly decreased the surface tension of 2.5% NaOCl and 2.5% Ca(OCl)_2_, as observed in a previous study using 2.4% NaOCl combined with benzalkonium chloride ^10^. Yet another prior study reported a reduced surface tension for 2.5% Ca(OCl)_2_ combined with benzalkonium chloride and cetrimide, although the authors used surfactant concentrations different from that corresponding to the CMC ^13^. In the present study, benzalkonium chloride, cetrimide, Tween 80 and Triton X-100 surfactants were added to 2.5% Ca(OCl)_2_ at CMC. The combination of surfactants could be an alternative to be considered. However, a study revealed that the combination of benzalkonium chloride, Triton X-100, ethyl formate and polyethylene glycol in 5.25% NaOCl might not be appropriate, since only benzalkonium chloride promoted the necessary reduction in surface tension ^21^.

The 2.5% Ca(OCl)_2_ solution showed lower surface tension than 2.5% NaOCl, which disagrees with studies that showed opposite results ^13^ ^,^ ^15^. The differences could be attributed to the methodology used, since the two studies used the ring method, whereas the present study used the pendant drop method. Although those studies were performed at 25°C room temperature and were in agreement regarding the surface tension of 2.5% Ca(OCl)_2_, they disagreed from each other regarding the surface tension of 2.5% NaOCl, namely: 64.68 mN/m ^15^ and 46.30 mN/m ^13^.

The measurement of free calcium ions is an important parameter to be considered, since it is involved in the mineralization repair processes by promoting osteoblastic and odontoblastic differentiation and mineralization of undifferentiated mesenchymal cells ^22^ ^,^ ^23^. All the surfactants increased the availability of free calcium ions in 2.5% Ca(OCl)_2_, but more pronouncedly for benzalkonium chloride. This can be explained by the cationic nature of the positively charged polar head of benzalkonium chloride ^12^, which may have induced repulsive interaction with the divalent calcium ion, thus hindering its binding to this ion. This effect was less pronounced in the case of Tween 80 and Triton X-100, which are non-ionic surfactants ^12^, and may interact with calcium ion in solution through ion-dipole interactions. The interaction and binding of calcium ion by hydroxyl groups has been described previously ^24^. In the case of cetrimide, its lower CMC compared with benzalkonium chloride may have influenced its behaviour in solution; however, more research is needed to substantiate this.

The crystal violet staining to assess penetration of the irrigants into dentine has been used previously ^1^ ^,^ ^9^ ^,^ ^25^ ^,^ ^26^. Both NaOCl and Ca(OCl)_2_ are chlorinated solutions containing the hypochlorite ion, a powerful oxidizing agent that bleaches the color of crystal violet, revealing the normal light color of dentine ^25^. In other words, since NaOCl and Ca(OCl)_2_ have to penetrate into the dentine to discolor it, the resultant bleached area can be correlated with penetration depth ^1^. The crystal violet staining has limitations such as the indirect assessment of the penetration through the bleached area, which is not necessarily correlated to antimicrobial activity, and the variable amount and diameter of dentinal tubules of each specimen, which may influenced the results. The contact time of the solutions in dentine was 2 min, as previously reported ^1^ ^,^ ^9^.

The 2.5% Ca(OCl)_2_ had a lower penetration depth into dentinal tubules than 2.5% NaOCl in the cervical and middle segments. The addition of benzalkonium chloride and Triton X-100 promoted a higher penetration of 2.5% NaOCl into dentinal tubules, in agreement with previous studies that used NaOCl with surfactants in those segments ^8^ ^,^ ^9^. Interestingly, the addition of surfactants did not improve the penetration of 2.5% Ca(OCl)_2_ into dentine. The low penetration of both Ca(OCl)_2_ and NaOCl with or without surfactant in the apical segment could have been influenced by the sclerotic dentine inherent in that segment ^27^.

To the best to our knowledge, there is no study that evaluated the penetration of 2.5% Ca(OCl)_2_ into dentine, hence precluding a proper comparison. The low penetration into dentinal tubules of Ca(OCl)_2_, with or without surfactants, could be attributed to the formation of calcium hydroxide precipitate when Ca(OCl)_2_ is at 25°C ^4^ ^,^ ^13^, hence obliterating the opening of dentinal tubules. Although the penetration depth of Ca(OCl)_2_ into dentinal tubules was lower than that of NaOCl, further research is needed to address the antimicrobial activity of Ca(OCl)_2_ along the extension of the dentinal tubules using confocal laser scanning microscopy. On the other hand, considering that the addition of surfactants led to a higher availability of free calcium ions, further research is also required to assess whether Ca(OCl)_2_ combined with surfactants has an effect on osteogenic and odontogenic differentiation of mesenchymal stem cells, rendering an outcome that could favour a repair process in endodontics.

## Conclusions

All the surfactants researched reduced the surface tension of NaOCl and Ca(OCl)_2_, without changing the pH or free available chlorine values, and allowed higher availability of free calcium ions in the Ca(OCl)_2_, especially benzalkonium chloride. Ca(OCl)_2_ had lower penetration into the dentinal tubules than NaOCl, while the benzalkonium chloride and Triton X-100 surfactants did not affect the penetration of Ca(OCl)_2_, but did increase the penetration of NaOCl.
